# Update on Nanoparticle-Based Drug Delivery System for Anti-inflammatory Treatment

**DOI:** 10.3389/fbioe.2021.630352

**Published:** 2021-02-17

**Authors:** Huailan Wang, Yunxiang Zhou, Qunan Sun, Chenghao Zhou, Shiyao Hu, Cameron Lenahan, Weilin Xu, Yongchuan Deng, Gonghui Li, Sifeng Tao

**Affiliations:** ^1^Department of Urology, Sir Run Run Shaw Hospital, School of Medicine, Zhejiang University, Hangzhou, China; ^2^Department of Surgical Oncology, The Second Affiliated Hospital, School of Medicine, Zhejiang University, Hangzhou, China; ^3^Department of Medical Oncology, The Second Affiliated Hospital, School of Medicine, Zhejiang University, Hangzhou, China; ^4^School of Medicine, Zhejiang University, Hangzhou, China; ^5^Center for Neuroscience Research, Loma Linda University School of Medicine, Loma Linda, CA, United States; ^6^Burrell College of Osteopathic Medicine, Las Cruces, NM, United States; ^7^Department of Neurosurgery, The Second Affiliated Hospital, School of Medicine, Zhejiang University, Hangzhou, China

**Keywords:** nanoparticle, anti-inflammatory, drug delivery, organic NPs, inorganic NPs, inflammatory disease

## Abstract

Nanobiotechnology plays an important role in drug delivery, and various kinds of nanoparticles have demonstrated new properties, which may provide opportunities in clinical treatment. Nanoparticle-mediated drug delivery systems have been used in anti-inflammatory therapies. Diseases, such as inflammatory bowel disease, rheumatoid arthritis, and osteoarthritis have been widely impacted by the pathogenesis of inflammation. Efficient delivery of anti-inflammatory drugs can reduce medical dosage and improve therapeutic effect. In this review, we discuss nanoparticles with potential anti-inflammatory activity, and we present a future perspective regarding the application of nanomedicine in inflammatory diseases.

## Introduction

The inflammatory response is a major pathogenic component in various diseases (Ferrero-Miliani et al., 2007). The innate immune response protects the host against inflammatory process, but also actives the innate inflammatory response system. Anti-inflammatory drugs help attain the balance of inflammatory and immune responses. Recent studies focus on hybrid materials that have anti-inflammatory effects and efficient drug delivery.

Currently, excessive production of inflammatory mediators contributes to the development of various diseases, including inflammatory bowel disease, rheumatoid arthritis, osteoarthritis (Lin et al., 2016), wound healing (Chang et al., 2020), and sepsis (Zhou et al., 2020). Several types of nanoparticles (NPs) reportedly have potential anti-inflammatory properties (Browne and Pandit, 2015).

Inflammatory bowel diseases (IBD), including Crohn’s disease and ulcerative colitis, are chronic intestinal inflammatory disorders (Zhang and Li, 2014). Moreover, the treatment requires frequent or continuous high-dosage administration of anti-inflammatory drugs. Nanoparticles with the ability to control drug release in the targeted location can assist with treatment and reduction of side effects (Lamprecht et al., 2001). Rheumatoid arthritis (RA) is a chronic auto-immune disease, and glucocorticoids (GCs) are considered a standard and efficient treatment for this disease. However, GC treatment is nonspecific and exerts systemic effects. Long-term treatments cause serious adverse reactions, but the current studies pertaining to nanoparticles describe a targeting drug delivery system thought to reduce systemic adverse reactions (Shi et al., 2020b).

Moreover, acute inflammatory disorders, such as sepsis (Zhou et al., 2020), trauma (Wang et al., 2019), and acute onset of chronic disease, are more complex than chronic inflammatory diseases. The treatments of these diseases require rapid and accurate drug delivery, but the nanoparticles are small enough to enrich the accumulation of drugs in the target tissue, consequently resulting in better treatment of diseases. In recent years, nanoparticle-based drug delivery systems have demonstrated potential in increasing anti-inflammatory effects and decreasing adverse effects. Moreover, novel drug delivery systems enhance the treatment of traditional drugs.

This review aims to list and compare different kinds of nanoparticle-based drug delivery systems with anti-inflammatory effects.

## Inflammation: Current Status and Treatment

Inflammation is a component of the non-specific immune response that occurs in reaction to harmful stimuli (Ferrero-Miliani et al., 2007). This definition implies that inflammation is an essential part of the disease process. Therefore, many diseases are associated with inflammation, and anti-inflammatory therapies are efficient in a plethora of diseases (Ma et al., 2017). Atherosclerotic vascular diseases are life-threatening diseases (Koga et al., 2016), as atherosclerosis is the cause of myocardial infarction, inflammation related disease (Katsuki et al., 2017) comprised of macrophage-mediated inflammation as the central mechanism (Hansson et al., 2006). Statins have been shown to have anti-inflammatory effects (Takemoto and Liao, 2001), the JUPITER (The Justification for the Use of Statins in Prevention: an Intervention Trial Evaluating Rosuvastatin) trial found that 44% of the major cardiovascular events can be decreased with intensive cholesterol lowering therapy (Ridker et al., 2008).

Tumors are the second leading cause of death in the world, and inflammation is a critical component of tumor progression (Coussens and Werb, 2002). The infiltration of leukocytes and inflammatory chemokines promote tumor development (Rollins, 2006). Interleukin-6 and tumor necrosis factor-α also promote the tumor metastasis (Qiao et al., 2016). The tumor inflammatory microenvironment is related with the immune system. Regarding tumor therapy, chemotherapy and immunotherapy remain common and effective treatments. However, limitations still exist in traditional therapy, such as low specificity, bone marrow suppression, and drug-resistance (Hori et al., 2018). After therapy, those dying cells stimulate inflammatory effects, reduce the therapeutic effect, and enhance drug-resistance. Currently, various NPs have been shown to have a targeting effect on tumor cells, nanoparticle-based drug delivery system improves the drug concentration in target system and reduces drug-resistance (Yao et al., 2020). Meanwhile, combined anti-inflammatory and anti-tumor drug delivery systems have been studied, and were found to be a potential tumor treatment, Huang et al., 2019) used red blood cell membrane (RBCm) vesicles as the shell to camouflage black phosphorus nanoparticle quantum dots (BPQDs) as drug carriers, Doxorubicin (OX) and Kirenol (KIR) were employed as anti-tumor and anti-inflammatory drugs. The nanoparticle platform RBC@BPQDs-DOX/KIR showed both anti-inflammatory and anti-tumor effect. However, such drug delivery systems with anti-inflammatory and anti-tumor properties still lacked research, and the stability and biological toxicity of the particles need further experimental verification.

Ocular disorders are a threat to patients’ vision (Stukenkemper et al., 2015). Diseases, such as xerophthalmia and allergic disease, affect many people, and intraocular implants have been used in intraocular defect diseases, glaucoma (Hoffman et al., 2002), and uveitis (Kempen et al., 2015). Most ocular diseases and surgeries (Johannesson et al., 2020) are related to an inflammatory response. The eye is a specific target site, and ocular medicine has become specialized to directly treat the eye. However, most cases of local inflammation that reach inner eye structures are treated via intraocular injections, but they have several limitations, such as drug bioavailability, local side effects, and unstable drug concentrations (Diebold and Calonge, 2010). Nanocarriers allow anti-inflammatory drugs to reach target structures. Moreover, different nano-size materials have different physicochemical features, which allow NPs to reach their target tissue. Chronic inflammatory diseases, such as age-related macular denegation (AMD) and uveitis, require the drug to be maintained at a certain concentration, which is important in treatment. However, biodegradable polymer carriers can cause intra ocular inflammation, Timo et al. synthesized self-assembling block copolypeptide NPs, these NPs were loaded with dexamethasone, which showed high loading efficiency and lasting cumulative drug release in eyes (Stukenkemper et al., 2015). Also, liposomes are biodegradable carrier which can improve the intimate contact between anti-inflammatory drug and corneal surface, allowing the improvement of ocular drug absorption (Chang et al., 2020). Dexamethasone, ibuprofen and other anti-inflammatory drugs loaded into these nanostructures improved drug efficiency and maintain a certain concentration into ocular tissue.

Sepsis is described as a life-threatening organ dysfunction caused by the dysregulated host response to infection (Singer et al., 2016), and is associated with high morbidity and mortality worldwide. However, the pathogenesis of sepsis remains unclear. When the body receives stimulation from bacteria, the immune system activates to attack the invasive bacteria. Part of the inflammatory process results from leukocytic adhesion and inflammatory cytokine-induced endothelial cell activation (Chacko et al., 2011; Phillipson and Kubes, 2011). Uncontrolled and excessive inflammatory responses cause sepsis. Regarding clinical treatment, antibiotics, and anti-inflammatory drugs are used to eliminate bacteria and control the inflammatory response. However, limitations still exist, such as severe adverse effects, dysfunction of liver and kidney, and poor bioavailability. Recent studies in nanomedicine have overcome some of these issues (Mitragotri et al., 2014; Torchilin, 2014). These NP pharmaceutical drug delivery systems (NDDSs) can be used to deliver antibiotics inside cells in the mouse model. Zhang et al. (2018) studied a co-delivery system of antibiotic and anti-inflammatory agents targeting infectious microenvironments, which managed sepsis in a mouse model. However, more studies are necessary to reveal the full potential of NDDSs.

Currently, inflammation-related diseases are more plentiful than above. Chronic diseases, such as IBD (Wu et al., 2019a), osteoarthritis (McMasters et al., 2017), rheumatoid arthritis (Shi et al., 2020b), and skin diseases, are treated with Non-Steroidal Anti-Inflammatory Drugs (NSAIDs) or Glucocorticoids as major or auxiliary treatment options, and frequent or continuous anti-inflammatory treatment is needed. Acute diseases, such as trauma, acute airway inflammation, and acute ischemic inflammation, are treated with high dosages of anti-inflammatory drugs (Shao et al., 2016; Xu et al., 2020). Furthermore, rapid and accurate treatment is needed. The development of pharmaceutical nanotechnology broadens the scope of drug delivery, as carriers assist in delivering the drug to target tissue, allowing release at an efficient range of concentration, which would significantly improve the accumulation of anti-inflammatory agents.

## Nanoparticle-Based Drug Delivery System in Anti-Inflammatory Treatment

Recently, several hybrid NPs have been studied in anti-inflammatory treatment. For better biocompatibility and targeting ability, current studies have mentioned that one or more materials are synthesized into nanostructures, and the characteristics of each material are fully utilized. Each structure has different feature that allow NPs to be placed in appropriate tissues. The synthesized NPs are used as carriers, and loaded with anti-inflammatory drugs to form a NPs-based drug delivery system.

### Organic NPs Based Drug Delivery System

Organic NPs show better biocompatibility, and most carriers are composed of biomaterial or polymer. These polymers are categorized as either natural or synthetic. Poly (lactic-co-glycolic acid) (PLGA) is a comparatively hydrophobic synthetic polymer (Bala et al., 2004), and has emerged as the most promising polymer used as a carrier in drug delivery, showing great potential in targeting and therapy (Mir et al., 2017), incorporating drugs into PLGA, forming a complex is widely studied. Thevenot et al. (2010) developed a mice implantation model and incorporated stromal cell-derived factor-1α (SDF- 1α) into PLGA. This solid scaffold reduced the inflammatory response when implanted into the subcutaneous space. Fredman et al. (2015) used an amino-terminal peptide encompassing amino acids 2-26 (Ac2-26), which can mimic annexin A1, and encapsulated Ac2-26 with collagen IV (Col IV)-targeted PLGA. Col IV-Ac2-26 PLGA NPs showed significant therapeutic efficacy in an atherosclerosis mice model. Regarding bone diseases, PLGA has been used in osteosarcomas, osteoarthritis, bone cancer metastasis, and other inflammatory bone diseases (Gu et al., 2013). Feng et al. (2010) incorporated doxycycline (DOXY) into PLGA nanospheres, formed a 3D scaffold, and then performed *in vitro* tests. Their group found that DOXY-PLGA NPs could inhibit the growth of *E. coli* and *S. aureus*, and released DOXY in a controlled manner. Peng et al. (2010) revealed a biodegradable thermosensitive implant composed of poly (ethylene glycol) monomethyl ether (mPEG) and PLGA as a solution-to-hydrogel (sol-gel) drug delivery system. The sol-gel drug delivery system was shown to have several advantages in osteomyelitis treatment, as demonstrated by using mPEG-PLGA, containing teicoplanin in the osteomyelitis rabbit model. Lastly, the histological examination and immunoblotting analysis found it effective in osteomyelitis treatment.

IBD is a group of chronic gastrointestinal diseases, including ulcerative colitis (UC) and Crohn’s disease (CD) (Yazeji et al., 2017). Tahara et al. (2011) found that oral decoy oligonucleotide (ODN) was convenient for UC mouse models. This study combined chitosan (CS)-modified PLGA nanospheres with nuclear factor kB (NF-kB) decoy ODN, forming an oral drug delivery system. Decoy ODN-loaded CS-PLGA NS improved ODN stability, but reduced bloody feces and diarrhea. The results showed that Decoy ODN-loaded CS-PLGA NS had potential to be an effective strategy for UC treatment. A lot of patients suffer from another common chronic inflammatory disease, rheumatoid arthritis. This condition is treated using low doses of methotrexate (MTX) and a disease-modifying anti-rheumatic drug (DMARD). Trujillo-Nolasco et al. (2019) found that Lutetium-177 (^177^Lu) could decrease synovial tissue inflammation. Moreover, 1,4,7,10-Tetraazacyclododecane-1,4,7,10-tetraacetic acid (DOTA) was used as an agent of ^177^Lu, which was synthesized using hyaluronic acid (HA) with ^177^Lu-DOTA and the complex was encapsulated with MTX and PLGA.^177^Lu-DOTA-HA-PLGA (MTX) demonstrated potential as a drug delivery system for anti-rheumatic therapy, but more *in vivo* models and tests are needed to corroborate the results.

Exosomes are 30–100 nm natural nanovesicles secreted by various cell types, such as tumor cells, mesenchymal stem cells, and immune cells. Exosomes are good carriers with low cytotoxicity, non-immunogenicity, and endogenous properties (Yan et al., 2020). Recently, exosome-based drug delivery systems have been studied, and it is worth noting that exosomes have a natural targeting property because of the native biological functions of the original cells, which may benefit disease treatment (Wu et al., 2020). Monocyte-derived myeloid cells have important roles in inflammatory diseases. Curcumin is a natural polyphenol extracted from the rhizomes of *Curcuma longa*, and has anti-inflammatory, antineoplastic, and antioxidant activities (Anand et al., 2008; Ravindran et al., 2009), but low systemic bioavailability (Deng et al., 2009). Sun et al. (2010) incorporated curcumin with exosomes, and then injected exosome-curcumin complex into a mice model. The complex protected mice against lipopolysaccharide (LPS)-induced septic shock. The exosome-curcumin complex enhanced the anti-inflammatory effect of curcumin, but the natural vehicle would not induce immune response, which avoided subsequent side effects. In colitis, the targeting ability of exosomes enabled accurate therapy (Yang and Merlin, 2019). Cai et al. (2012) found that exosomes derived from the TGF-β1 gene-modified BMDC (TGF-β1-EXO) could effectively inhibit the development of dextran sulfate sodium (DSS)-induced murine IBD. Moreover, the protective ability was dosage-related. Wang et al. (2016) isolated exosomes from the culture supernatant of granulocytic myeloid-derived suppressor cells (G-MDSC), and found that G-MDSC exosomes could attenuate DSS-induced colitis to restore intestinal immune balance. Wu et al. (2020) studied the molecularly engineered M2 macrophage-derived exosome (m2 exo). They selected hexyl 5-aminolevulinate hydrochloride (HAL) to obtain HAL-containing M2 exosome (HAL@M2 Exo) and found that HAL@M2 Exo had excellent inflammation-tropism capability, as both *in vitro* and *in vivo* experiments demonstrated anti-inflammatory effects in atherosclerosis. Yan et al. (2020) used exosome as carrier, encapsulating dexamethasone sodium phosphate, modified the surface with folic acid (FA)-polyethylene glycol (PEG)-cholesterol (Chol), *in vitro* study, the FPC-Exo/Dex drug delivery system showed anti-inflammatory effect against RAW264.7 cells.

Liposomes are the first closed bilayer phospholipid system, and lipidic nanoparticles are the first nanomedicine delivery system used in clinical application for various tumors (Allen and Cullis, 2013). Currently, anti-inflammatory effects of liposomal nanoparticles have been studied (Chiong et al., 2013; Chang et al., 2020). Interleukin-10 (IL-10) is a cytokine that can reduce the production of pro-inflammatory cytokines (Takeda et al., 1999). IL-10 administration is effective for several inflammatory diseases, such as IBD, rheumatoid arthritis, and organ transplantation (Asadullah et al., 2003). Toita et al. (2016) used liposomes containing phosphatidylserine (PS) (PSL) as a biomaterial carrier. They encapsulated IL-10, and this IL-10-conjugated PSL (PSL-IL 10) showed significant anti-inflammatory and anti-obesity effects in an obese mice model. Moreover, PSL mimics apoptotic cells, changes inflammatory M1 macrophages to anti-inflammatory M2 macrophages, and can be specifically recognized by macrophages (Nagata et al., 2010). Wu et al. (2019b) utilized the “eat me” signal of PS, modified liposomes with PS and DSPE-PEG2000-cRGDfK, formed an apoptotic body liposome (AP-Lipo), and then loaded with pioglitazone (PIO). The study found that AP-Lipo was more effective in recognizing the activated vascular endothelial monolayer and upregulating anti-inflammatory cytokines *in vitro*. PIO-loaded AP-Lipo demonstrated ability to target atherosclerotic plaque, and the anti-inflammatory effect was investigated by inhibiting M1 polarization and promoting M2 macrophage polarization. Xu et al. (2019) modified polyetheretherketone (PEEK) with dexamethasone plus minocycline-loaded liposomes (Dex/Mino liposomes). *In vitro* and *in vivo* experiments showed an enhanced anti-inflammatory, antibacterial, and osseointegrative capacity of this hybrid nanoparticle, which showed great potential as an orthopedic/dental implant nanomaterial for clinical application. Generally, nanoparticles, such as liposome, containing PS, have been used to mimic cellular debris. Numerous models had proven that PSL has a direct effect on anti-inflammatory cytokine production, and showed potential as an anti-inflammatory and immunomodulatory agent (Bagalkot et al., 2016).

Excluding the nanomedicines above, carriers, such as collagen hydrogel (Wang et al., 2014), gelatin hydrogel (Ratanavaraporn et al., 2012), lactide-co-glycoside (PLG) scaffold (Gower et al., 2014), Hyaluronic acid hydrogel (Nakamura et al., 2004), Amphiphilic poly-N-vinylpyrrolidone (Amph-PVP) (Kuskov et al., 2017), and Emulgel (Gul et al., 2018), influence the biocompatibility and efficiency of anti-inflammatory effects, as well as enhance drug delivery accuracy. Different structures of these drug delivery systems target various tissues. Some studies focus on macrophages, as most inflammation is related to macrophages, which regulate pro-inflammatory and anti-inflammatory effects. Therefore, macrophages are key targets for treating inflammation (Mosser and Edwards, 2008; Table 1).

**TABLE 1:** The application of organic nanoparticle-based drug delivery system.

**Drug**	**Carrier**	**Disease**	**Structure**	**References**
SDF- 1α	PLGA	Implant station	Solid scaffold	Thevenot et al., 2010
Ac2-26	Col IV-targeted PLGA	Atherosclerosis	Nanoparticle	Fredman et al., 2015
DOXY	PLGA	Periodontitis and arthritis	3D scaffold	Feng et al., 2010
Teicoplanin	mPEG-PLGA	Osteomyelitis	Sol-gel	Peng et al., 2010
ODN	CS-PLGA	Ulcerative colitis	Nanospheres	Tahara et al., 2011
MTX	^177^Lu-DOTA-HA-PLGA	Rheumatic arthritis	Nanoparticle	Trujillo-Nolasco et al., 2019
Curcumin	Exosome	Septic shock	Nanoparticle	Sun et al., 2010
Dex	FPC-Exosome	Rheumatic arthritis	Nanoparticle	Yan et al., 2020
NA	TGF-β1-Exosome	Colitis	Nanoparticle	Cai et al., 2012
NA	G-MDSC Exosome	Colitis	Nanoparticle	Wang et al., 2016
HAL	M2 Exosome	Atherosclerosis	Nanoparticle	Wu et al., 2020
IL-10	Liposome	Obesity	Nanoparticle	Toita et al., 2016
PIO	Liposome	Atherosclerotic plaques	Nanoparticle	Wu et al., 2019b
Dex/Mino	Liposome-PEEK	Orthopedic/dental implant	Nanoparticle	Xu et al., 2019

*Ac2-26, amino acids 2-26; Col IV, collagen IV; CS, chitosan; DOXY, doxycycline; Dex, dexamethasone; Dex/Mino, dexamethasone plus minocycline; FPC, folic acid (FA)-polyethylene glycol (PEG)-cholesterol (Chol); G-MDSC, granulocytic myeloid-derived suppressor cells; HAL, hexyl 5-aminolevulinate hydrochloride; 177Lu-DOTA-HA-PLGA, Lutetium-177-1,4,7,10-Tetraazacyclododecane-1,4,7,10-tetraacetic acid-hyaluronic acid-poly (lactic-co-glycolic acid); IL-10, Interleukin-10; mPEG, poly (ethylene glycol) monomethyl ether; NA, not available; ODN, oral decoy oligonucleotide; PLGA, Poly (lactic-co-glycolic acid); PIO, pioglitazone; SDF- 1α, stromal cell-derived factor-1α; Sol-gel, solution to hydrogel.*

### Inorganic NPs Based Drug Delivery Systems

Inorganic NPs used to treat inflammation are commonly constituted with anti-inflammatory drugs and inorganic carriers. Metal oxide NPs are more stable, and demonstrate far-ranging prospects in nanomedicine as a vehicle for drug delivery. The zinc oxide nanoparticle (ZnO NPs) has excellent biomedical properties (Agarwal and Shanmugam, 2020), as Zn plays an important role in the transmission of genetic messages (Auld, 2001). ZnO is a very strong antibacterial agent, and these ZnO nanoparticles have proven bacteriostatic properties. Studies have been done to find the mechanism, and a possible explanation is that the electrostatic interaction between ZnO and the cell wall destroyed the integrity of the bacterial cell (Brayner et al., 2006), allowing Zn^2+^ ions to penetrate into bacterial cells (Brunner et al., 2006), forming ROS reaction (Lipovsky et al., 2011). The free radicals induced membranal damage and interacted with DNA, leading to cell death (Buszewski et al., 2018). Based on the antibacterial effect of ZnO, various nanoparticles have been studied. One study found the targeted release of drugs by folic acid-modified PEG-ZnO NPs showed anti-inflammatory and anti-tumor co-effect in drug delivery (Vimala and Soundarapandian, 2017), and another study found the controlled release of doxorubicin (DOX) hydrochloride by mesoporus ZnO NPs also showed anti-inflammatory and anti-tumor co-effect (Barick et al., 2010), but more *in vitro* and *in vivo* experiments are necessary to study the advantage of ZnO as a carrier in drug delivery. Meanwhile, the gold nanocage (GNC) is proven to be an ideal drug delivery system (Dul et al., 2019). Shi et al. (2020a) used aspirin-containing GNCs (As@GNCs) to continuously stimulate and initiate an immune response for monocyte recruitment, facilitating the internalization of aspirin-laden monocytes (AsMon). Then, As@GNC-laden monocytes could target the infection area and differentiate into bactericidal macrophages. This anti-inflammatory effect was demonstrated in the MRSA-induced osteomyelitis mice model. Gold nanoparticles (GNP) have unique properties for improved therapy. Gao et al. (2019) identified a peptide-GNP hybrid, P12 (G20), with a GNP core of 20 nm, which showed potent activity in reducing Toll-like receptor (TLR) signaling, which was the key factor in the inflammatory response of acute lung injury (ALI). Moreover, P12 (G20) inhibited inflammation *in vivo* in the LPS-induced ALI mouse model. Bare ZnO NPs and GNPs are unstable and prone to aggregation, which limits their application. Previously, the metal NPs were modified with polymers or peptides, or their structures were changed to improve biostability and provide further application.

The Quantum dot (QD) is a fluorescent semiconductor nanoparticle that has been widely studied in theranostic application. Besides, current studies have shown that QD has potential to participate in drug delivery. NSAIDs are anti-inflammatory molecules used to treat inflammatory disorders. Kalangi et al. (2018) linked celecoxib with QD. These QD-Celecoxib conjugates clearly targeted the inflamed paw of the mice, not only exerting an accurate anti-inflammatory effect, but a bio-imaging effect was also observed. Sameer Kumar et al. (2018) combined sodium 10-amino-2-methoxyundecanoate (SAM) with N-doped graphene quantum dots (N-GQDs) in an *in vitro* experiment. SAM combined with N-GQDs improved downregulation of COX-2, iNOS, TNF-α, NF-κß, IL-1α, IL-1ß, IL-4, and IL-6 compared with cells treated with SAM alone. With these modulations, SAM combined with N-GQD was thought to have effective anti-inflammatory potential.

In recent decades, there have been thousands of studies focusing on the synthesis and application of Carbon nanotubes (CNTs) (Jia and Wei, 2017), which gained attention to be promising nanocarrier (Wong et al., 2013). CNTs may be paired with anti-inflammatory drugs, and act as the main drug vehicle or adjunct to assist or modify drug release from another parent delivery system. NSAIDs are commonly used to alleviate arthritic pain, are usual routes of administration include transdermal and oral delivery. Transdermal administration often causes low concentration and lacks precise control, while oral administration often produces systemic side effects. CNTs have been used to modulate and enhance the release of NSAIDs at a relatively low voltage, which is non-irritating to skin (Spizzirri et al., 2013). Im et al. (2010) used multi-walled carbon nanotubes (MWCNTs) as an additive to reinforce the hydrogel matrix. Water-soluble poly-ethylene oxide (PEO) and water-insoluble Pentaerythritol triacrylate (PETA) formed the network, ketoprofen was electrospun into fibers, and all above constituted a transdermal drug delivery system with biocompatibility. The CNTs accelerated the effect of electric voltage, leading to increased drug release and facilitation of the anti-inflammatory effect (Table 2).

**TABLE 2:** The application of inorganic nanoparticle-based drug delivery system.

**Drug**	**Carrier**	**Disease**	**Structure**	**References**
Monocyte	As@GNC	Osteomyelitis	Nanoparticle	Shi et al., 2020a
Celecoxib	QD	Local edema	Nanoparticle	Kalangi et al., 2018
SAM	N-GQD	NA	Nanoparticle	Sameer Kumar et al., 2018
Diclofenac sodium	CNT	Arthritic pain	Nanoparticle	Spizzirri et al., 2013
Ketoprofen	PEO/PETA-MWCNT	NA	Nanotube	Im et al., 2010

*As@GNC, aspirin-containing gold nanocage; CNT, Carbon nanotube; NA, not applicable; PEO/PETA-MWCNT, poly-ethylene oxide/Pentaerythritol triacrylate-multi-walled carbon nanotubes; QD, Quantum dot; SAM, sodium 10-amino-2-methoxyundecanoate.*

## Conclusion and Future Direction

Inflammation includes bacterial inflammation and non-bacterial inflammation, and is commonly treated using steroidal and non-steroidal anti-inflammatory drugs, paired with antibiotics in bacteria-related inflammation. Commonly, chronic inflammations may cause systemic disorders, acute inflammation may develop into a serious condition, and even become life-threatening. Traditional treatments have limitations, such as systemic adverse effects, inadequate local drug concentration, gastrointestinal ulcer formation, unstable continuous administration, and difficulty in maintaining drug concentration.

In recent years, nanomedicine has become a multidisciplinary field that promotes physicochemical and biological constituents of nanoparticles. Nanotechnology has been widely used and fully developed in anti-inflammatory therapy. Whether it is organic nanoparticles or inorganic nanoparticles, the advantages of various materials establish new approaches to enhance therapeutic effects while reducing adverse effects. This review has listed most nanoparticle-based drug delivery systems above. Some remain in the basic science phase, whereas some of the organic NPs have already been used in clinical treatment. The proposed nanoparticle-based drug delivery systems must be biocompatible, biodegradable, non-toxic, and must have controlled release of drug. Hence, carriers and assists are actually major components in forming an appropriate drug delivery system.

With the exception for improving the target effect and bioavailability of vehicle or assist, there will be wider choice for drugs in the future. Chinese herbs are currently under study. Markus et al. (2017) had studied the anti-inflammatory effect of the *Angelica pubescens* root. This plant, also known as Du Huo (DH), was combined with gold nanoparticles (DH-AuNPs). DH-AuNPs demonstrated favorable application to sites of inflammation and drug release to the accurate site. The Chinese herb is very complex and the mechanism remains unclear. However, further studies are warranted to confirm this Chinese herb’s potential as an ideal anti-inflammatory drug. Moreover, Quantum dot shows strong targeting and bio-imaging abilities, and size variation allows it to be modified by various peptides, inorganic materials, and antibodies to be used for accurate, targeted therapy for inflammation, particularly immune-related inflammation. The potentiality of QD is not only represented in anti-inflammatory therapy, but also in real-time positioning and imaging, which can compound the treatment effect.

Although NPs showed multiple advantage in drug delivery, there are still various shortcomings need to be resolved before clinical application. The comparison between different type of NPs is shown in Table 3, PLGA was widely used in vehicle synthesis, for its variable structure and stability, however, PLGA can cause new inflammation *in vivo*, showed little toxicity in liver and intestine in F344 rat (Navarro et al., 2016). The biocompatibility needs to be improved, current studies modified PLGA with HA, chitosan, and other materials, which could be the future direction to improve PLGA-based drug delivery system. Exosomes are vehicles secreted from tissues, with good biocompatibility and targeting ability, different sizes of exosome can be collected, it is different to purify exosome, improving the uniformity of exosome is the key to better use of their advantages. Liposome has been used in clinical for drug delivery, most drug toxicities have been reduced when they are coated with liposome, however, it is not the best vehicle for water-soluble drugs. As for metal carrier, ZnO NPs and GNP are not stable, which limit their application. CNT is a promising vehicle with good biocompatibility, however, the targeting ability needs to be improved. QDs are now widely studied in bio-imaging, most QDs contain heavy metal, reduce the biological toxicity and maintain its targeting and bio-imaging ability are future direction in drug delivery. A better understanding of anti-inflammatory diseases and targeted use of NPs for different disease will fundamentally improve the therapeutic effect and reduce the side effects of drugs.

**TABLE 3:** Comparison between organic nanoparticles (NPs) and inorganic NPs.

**Types**	**Carrier**	**Advantages**	**Disadvantages**
Organic NPs	PLGA	Multiple structures with good stability	Biocompatibility needs to be improved
	Exosome	Organic-targeted effect, sufficient source	Difficult to obtain high purity exosome, poor uniformity
	Liposome	Easy to obtain, good stability, biodegradable	Unstable for water-soluble drugs, short storage time
	……		
Inorganic NPs	Au/Zn	Antibacterial or bacteriostatic property	Unstable
	CNT	Good Biocompatibility	Poor targeting ability
	QD	Targeting and bio-imaging ability	Biological toxicity
	……		

*NPs, nanoparticles (NPs); PLGA, Poly (lactic-co-glycolic acid); CNT, Carbon nanotube; QD, Quantum dot.*

## Author Contributions

YZ conceptualized the research project. HW and QS drafted the manuscript. YZ, QS, WX, and ST reviewed and modified the manuscript. HW and SH made the tables. ST, YD, GL, and CL supervised the research, led the discussion. All authors approved the final version of the manuscript.

## Conflict of Interest

The authors declare that the research was conducted in the absence of any commercial or financial relationships that could be construed as a potential conflict of interest.

## Abbreviations

Ac2-26: amino acids 2-26
ALI: acute lung injury
AMD: age-related macular denegation
Amph-PVP: amphiphilic poly-N-vinylpyrrolidone
AP-Lipo: apoptotic body liposome
As@GNC: As@GNC
AsMon: aspirin-laden monocytes
CD: Crohn’s disease
CNTs: carbon nanotubes
Col IV: collagen IV
CS: chitosan
Dex: dexamethasone
DH-AuNP: Du Huo combined with gold nanoparticle
DMARD: disease-modifying anti-rheumatic drug
DOTA: 1,4,7,10-Tetraazacyclododecane-1,4,7,10-tetraacetic acid
DOX: doxorubicin
DOXY: doxycycline
DSS: dextran sulfate sodium
FPC: folic acid (FA)-polyethylene glycol (PEG)-cholesterol (Chol)
GCs: glucocorticoids
G-MDSC: granulocytic myeloid-derived suppressor cells
GNC: gold nanocage
GNP: Gold nanoparticle
HA: hyaluronic acid
HAL: hexyl 5-aminolevulinate hydrochloride
IBD: inflammatory bowel diseases
IL: interleukin
LPS: lipopolysaccharide
mPEG: poly (ethylene glycol) monomethyl ether
MTX: methotrexate
MWCNTs: multi-walled carbon nanotubes
NDDS: nanoparticulate pharmaceutical drug delivery system
NPs: nanoparticles
NF- κ ß: nuclear factor κ ß
N-GQD: N-doped graphene quantum dot
NSAIDS: non-steroidal anti-inflammatory drugs
PEEK: polyetheretherketone
PEO: poly-ethylene oxide
PETA: pentaerythritol triacrylate
PIO: pioglitazone
PLG: lactide-co-glycoside
PLGA: poly (lactic-co-glycolic acid)
PS: phosphatidylserine
PSL: liposomes containing phosphatidylserine
QD: quantum dot
RA: rheumatoid arthritis
SAM: sodium 10-amino-2-methoxyundecanoate
SDF-1 α: stromal cell-derived factor-1 α
sol-gel: solution-to-hydrogel
TLR: Toll-like receptor
UC: ulcerative coliti
ZnO: Zinc oxide nanoparticle.
